# Current Applications of Artificial Intelligence in Benign Prostatic Hyperplasia

**DOI:** 10.5152/tud.2022.22028

**Published:** 2022-07-01

**Authors:** Milap Shah, Nithesh Naik, BM Zeeshan Hameed, Rahul Paul, Dasharathraj K Shetty, Sufyan Ibrahim, Bhavan Prasad Rai, Piotr Chlosta, Patrick Rice, Bhaskar K Somani

**Affiliations:** 1 Robotics and Urooncology, Max Hospital and Max Institute of Cancer Care, New Delhi, India; 2 iTRUE (International Training and Research in Uro-oncology and Endourology) Group, Manipal, Karnataka, India; 3 Department of Mechanical and Manufacturing Engineering, Manipal Institute of Technology, Manipal Academy of Higher Education, Manipal, Karnataka, India; 4 Department of Urology, Father Muller Medical College, Mangalore, Karnataka, India; 5 Department of Radiation Oncology, Massachusetts General Hospital, Harvard Medical School, Boston, USA; 6 Department of Humanities and Management, Manipal Institute of Technology, Manipal Academy of Higher Education, Manipal, Karnataka, India; 7 Kasturba Medical College, Manipal Academy of Higher Education, Manipal, Karnataka, India; 8 Department of Urology, Freeman Hospital, Newcastle, UK; 9 Department of Urology, Jagiellonian University in Krakow, Kraków, Poland; 10 Department of Urology, University Hospital Southampton NHS Trust, Southampton, UK

## Abstract

Artificial intelligence is used in predicting the clinical outcomes before minimally invasive treatments for benign prostatic hyperplasia, to address the insufficient reliability despite multiple assessment parameters, such as flow rates and symptom scores. Various models of artificial intelligence and its contemporary applications in benign prostatic hyperplasia are reviewed and discussed. A search strategy adapted to identify and review the literature on the application of artificial intelligence with a dedicated search string with the following keywords: “Machine Learning,” “Artificial Intelligence,” AND “Benign Prostate Enlargement” OR “BPH” OR “Benign Prostatic Hyperplasia” was included and categorized. Review articles, editorial comments, and non-urologic studies were excluded. In the present review, 1600 patients were included from 4 studies that used different classifiers such as fuzzy systems, computer-based vision systems, and clinical data mining to study the applications of artificial intelligence in diagnoses and severity prediction and determine clinical factors responsible for treatment response in benign prostatic hyperplasia. The accuracy to correctly diagnose benign prostatic hyperplasia by Fuzzy systems was 90%, while that of computer-based vision system was 96.3%. Data mining achieved sensitivity and specificity of 70% and 50%, respectively, in correctly predicting the clinical response to medical treatment in benign prostatic hyperplasia. Artificial intelligence is gaining attraction in urology, with the potential to improve diagnostics and patient care. The results of artificial intelligence-based applications in benign prostatic hyperplasia are promising but lack generalizability of results. However, in the future, we will see a shift in the clinical paradigm as artificial intelligence applications will find their place in the guidelines and revolutionize the decision-making process.

**Keywords: **Artificial intelligence; deep learning; machine learning; benign prostate enlargement; benign prostatic hyperplasia.

**Main Points**

- Artificial intelligence (AI)-based systems have gained significant attraction in various urological subspecialties.
- Artificial intelligence has been particularly useful in screening, symptom analysis, investigation, and management of benign prostate hyperplasia (BPH).
- It has improved the notion of predicting clinical outcomes before surgical intervention thus aiding the surgeon to make better-informed decisions.
- In this review of over 1600 patients from 4 studies, Fuzzy systems could accurately diagnose 90% of BPH cases. while computer-based vision systems diagnosed 96.3% of the cases.
- Predicting the clinical response to medical treatment in BPH, data mining achieved sensitivity and specificity of 70% and 50%, respectively.

## Introduction

Benign prostatic hyperplasia (BPH) affects over 200 million men worldwide and is the leading cause of lower urinary tract symptoms (LUTS) in aging men.^1,2^ Decreased flow rates along with an increase in bothersome voiding and storage symptoms are all features of untreated BPH, which can lead to acute or chronic urinary retention.^2^ The existing diagnostic methods of BPH cannot cope with the issues such as improper, uncertain hesitant responses of patients. Furthermore, existing evaluation approaches may lose some useful responses to result in the indeterminate diagnosis in the evaluation process of these patients.^3^ In terms of predicting the clinical outcomes before minimally invasive treatments for BPH, there is insufficient reliability despite multiple assessment parameters, such as flow rates and symptom scores.^4^ Many questionnaires are available for the clinical prediction of BPH, yet the results can be unreliable and inaccurate.^5^

**Figure 1.**
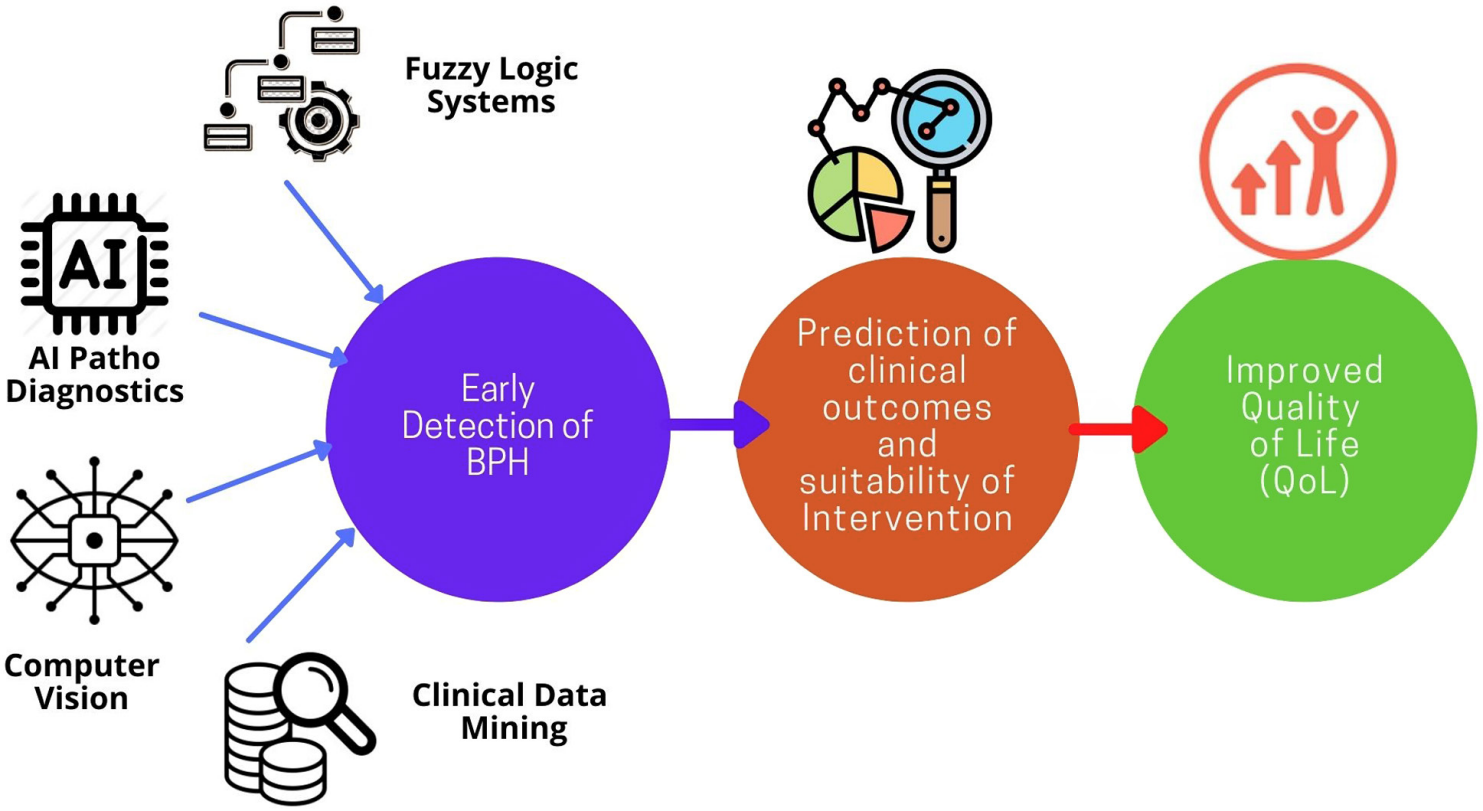
Current applications of artificial intelligence in benign prostatic hyperplasia (BPH).

Artificial intelligence (AI) refers to the computational capability of the machine to mimic and perform human cognitive tasks. It is causing a paradigm shift in terms of providing health care and decision-making for clinicians.^6^ The advances in medical technologies used in health care, such as electronic medical records, are providing humongous amounts of data.^7,8^ Artificial intelligence provides more accuracy and reliable cues for clinical decisions; hence, it is possibly going to be an integral part of the health care system.^8^ Figure 1 shows the current applications of AI in BPH. Artificial intelligence methodologies are more accurate in prediction and more explorative for studying big data cohorts than traditional statistics, and it has also been widely utilized in urology.^9^ Artificial intelligence has aided evidence-based and personalized medical treatment by making a growing collection of patient data available to physicians.^10^

While the notion that early diagnosis of BPH is meaningful in delaying disease progression has been largely accepted, there are limited objective investigations that are operator-independent such as the transrectal ultrasound (TRUS) which has significant inter-observer variability. There have been attempts to standardize this with the help of AI to fulfill clinical expectations.^11^ With the increasing use of predictive models that combine in a non-linear manner, several predictors have also been established to differentiate BPH from prostate cancer (PCa).^11,12^ Along with understanding the usefulness of predicting clinical outcomes before performing minimally invasive therapeutic surgeries in BPH, there is also a growing trend of AI aiding the surgeon to make better-informed decisions.^13^ Therefore, there is increasing uptake of utilizing AI in screening, symptom analysis, investigation, and management of BPH like other urological pathologies. The present systematic review aims to give a comprehensive summary of the contemporary applications of AI in BPH.

### Search Strategy and Article Selection

A search strategy was adapted to identify and review the literature on the application of PubMed, ScienceDirect, Embase, Medline, and CINAHL. The search strategy was conducted according to the Patient–Intervention–Comparison–Outcome criteria where patients with BPH (P) whose conditions were managed with AI tools (I) or traditional biostatistical models (C) were examined to evaluate the efficacy of AI models (O). A dedicated search string was then created based on a combination of the following keywords: “Machine Learning,” “artificial intelligence,” AND “benign prostate enlargement” OR “BPH” OR “Benign prostatic hyperplasia.”

Articles were selected according to the Preferred Reporting Items for Systematic Reviews and Meta-analyses (PRISMA) criteria.^14^

### Inclusion Criteria

1. Articles on BPH and AI;
2. Full-text original articles on all aspects of diagnosis, treatment, and outcomes of BPH.

### Exclusion Criteria

1. Editorials, commentaries, abstracts, reviews, or book chapters;
2. Animal, laboratory, or cadaveric studies.

Only original articles in English were included from inception to January 2021. Editorials, commentaries, abstracts, reviews, book chapters, and studies reporting experimental studies on animals or cadavers were excluded from the review. The literature review was performed according to the inclusion and exclusion criteria. The references list of the selected articles was individually and manually reviewed to screen for additional articles of interest.

### Evidence Synthesis

**Figure 2.**
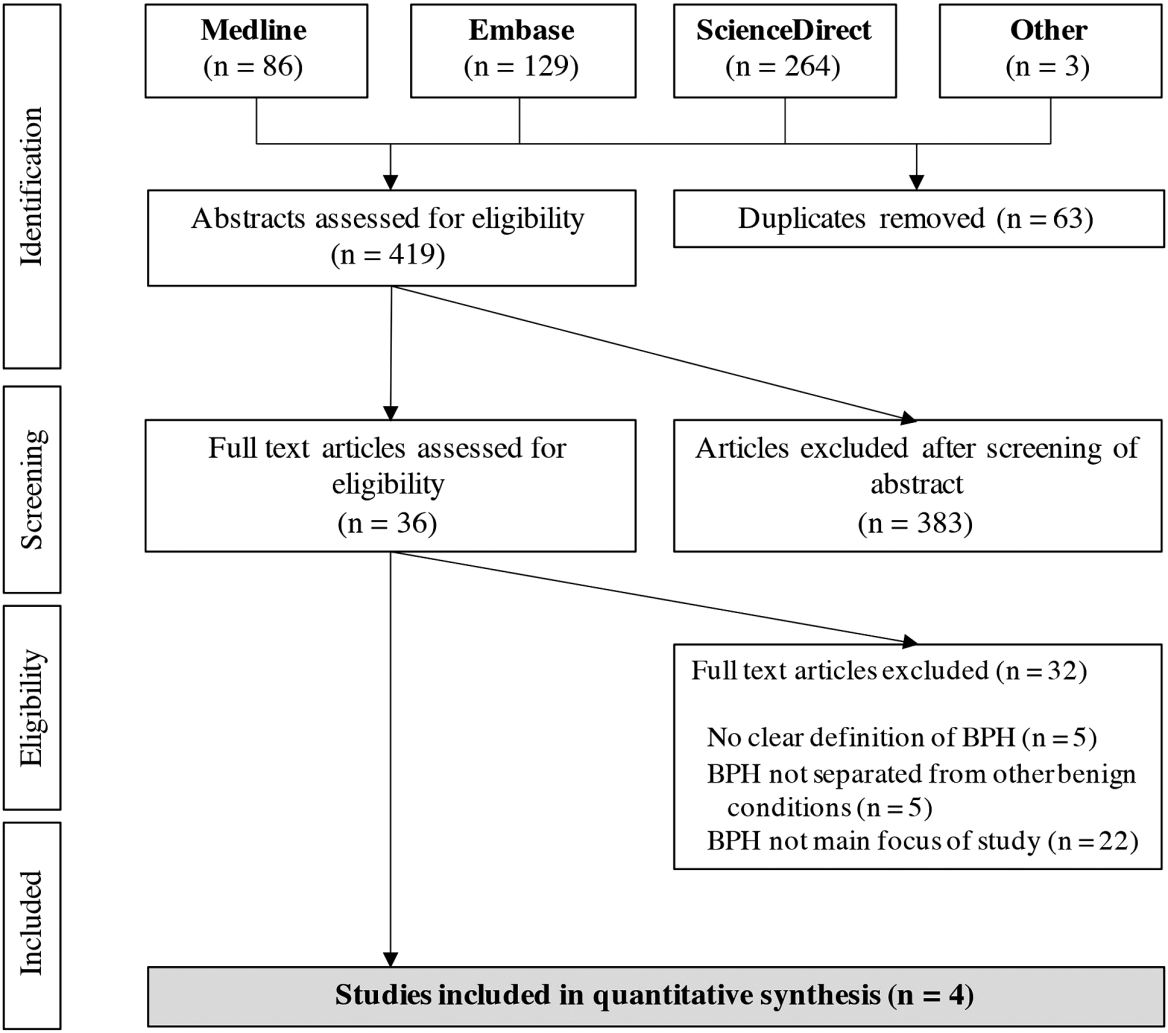
Preferred Reporting Items for Systematic Reviews and Meta-analyses flow diagram.

The initial search identified a total of 482 articles. From this list, 419 articles were assessed for eligibility following the removal of duplicate articles (n = 63). After additional screening and review of these articles, a total of 4 articles were identified that met our inclusion criteria and were subsequently included in the final review as per PRISMA (Figure 2).

### Applications of AI in Benign Prostatic Hyperplasia

There is a great motivation for researchers to develop intelligent systems to get various signs and symptoms of the patient and determine the level of the disease severity intelligently among patients with BPH. However, very limited research has been done in this area. In this article, we shall highlight key features of the following studies done using various AI-based applications in diagnosis, decision-making, as well as differentiation from prostate cancer. A summary of the included studies is reported in Table 1.^3,15-17^

Fu et al^3^ used the cubic hesitant fuzzy sets (CHFS) and the dice measure of CHFSs for the initial diagnosis and evaluation of BPH and compared them with the existing evaluation methods. The main advantage of this system is that it can accurately assess even when the objective information provided by the patient is uncertain and hybrid. Torshizi et al^15^ used fuzzy intelligent systems with 2 modules to diagnose the severity level of BPH and recommend treatment for the same in diagnosing severity level and appropriate therapies for patients (n = 44). The first module determined the degree of severity and the second module recommended a treatment decision based on ontology modeling which used the results of the first module and added external knowledge as well. To validate the efficiency and accuracy of the developed system, a case study was conducted on 44 participants. The results were then compared with the decisions and recommendations of an expert panel. The model achieved an accuracy of 90%, suggesting that decisions suggested by the fuzzy system and expert panel matched in 90% of these cases.

Khalid et al^16^ evaluated the application of a computer vision-based system for the histopathological diagnosis of BPH using 59 digital histopathological microphotograph pictures divided into training (70%), validation (10%), and test (20%) dataset. In these 59 images, 169 regions were marked as sites of hyperplasia of the prostatic glandular component. In each dataset, the regions of hyperplasia were marked in the images. The model achieved an accuracy of 96.3% incorrectly identifying the hyperplastic glandular areas.

Fusco et al^17^ designed an integrated clinical data mining analysis system to identify factors associated with a clinically meaningful response to placebo or tadalafil 5 mg once daily in patients with LUTS–BPH. The dataset of 1500 patients was randomly split into training and test subsets. Logistic regression (LR), random forest (RF), decision tree (DT), support vector machine (SVM), and models were then generated on the training subset and used to predict response in the test subset. The cut-off was 80% for sensitivity and specificity to differentiate between those responding to treatment and those who did not. This model achieved the highest combined sensitivity and specificity of 70% and 50%, respectively. This model did not identify baseline characteristics that could predict individual patient response to placebo or once-daily tadalafil. However, the study reaffirms the efficacy of tadalafil in the treatment of LUTS–BPH in the majority of patients and the importance of evaluating individual patient need in selecting the most appropriate treatment.^17^

## Results

In the present review, 1600 patients were included from 4 studies as mentioned above which used different classifiers such as fuzzy systems, computer-based vision systems, and clinical data mining to study the applications of AI in diagnoses, severity prediction, and determine clinical factors responsible for treatment response in BPH. The accuracy to correctly diagnose BPH by Fuzzy systems was 90%, while that by computer-based vision system was 96.3%. Data mining achieved sensitivity and specificity of 70% and 50%, respectively, in correctly predicting the clinical response to medical treatment in BPH.

### Strengths, Limitations, and Areas of Future Research

The use of a wide variety of AI models and algorithms did not allow us to pool the data together. However, we have included all AI-related BPH articles and summarized their current clinical use and role within this clinical spectrum. Artificial intelligence has been used in all areas of BPH including diagnosis, as well as for predicting treatment response and success. However, it is still a research-based tool and is not used universally in clinical practice. A lacuna in the application of intelligent systems in the diagnosis and decision-making of BPH is visible. However, the role of such intelligent systems in this condition is debatable. This could be due to a lack of data infrastructure needed to train the algorithms, wider applicability in all groups of patients, complexity of its use, and cost involved with it.^18,19^

Another limitation is the lack of application of deep convolutional neural networks (DCCN) for the diagnosis and classification of BPH, though, studies have been conducted on DCCN to differentiate between PCa and BPH.^20-22^ Wong et al^20^ used deep-learning-based networks to detect PCa in transitional zone (TZ) and differentiate it from BPH using T2-weighted (T2W) and apparent diffusion coefficient (ADC) map MR images. Using dataset of 196 patients, the model using ADC alone demonstrated higher sensitivity (0.829) and precision (0.534) as compared to T2W and T2W + ADC models.^20^ A similar study was conducted by Hu et al^21^ using deep transfer learning (DTL) methods to overcome the disadvantage of smaller datasets. They concluded that DTL methods are particularly useful in developing regions where multiparametric MRI is not easily available and the accurate diagnosis of PCa in TZ can be achieved via DTL from disease-related images. Mehralivand et al used a 3-dimensional U-Net-based DCNN algorithm for automated detection and segmentation of prostate MRI lesions and predict Prostate Imaging Reporting and Data System (PI-RADS) categories 2-5 and BPH. The model achieved 56.1% sensitivity and 62.7% positive predictive value in the detection of lesions including BPH and overall PI-RADS classification accuracy of 30.8% suggesting a reasonable detection and classification performance metrics.^22^ Studies can be conducted in the future on similar lines to solely diagnose, classify, as well determine the severity of BPH using radiomics and DCCN. AI studies conducted in the future should also focus more on quality of life, suitability of the various treatment options, as well as the cost of treatment, and come up with common algorithms that can be used universally. A surgical safety monitoring system can also be developed in the future based on this method to warn the surgeons about complications such as incontinence and bleeding during the procedure and improve patient safety. While the use of AI has increased in all urological sub-specialties, it is time to standardize outcome measures to help compare treatment modalities and make individualized patient-centered decisions.^23-25^ This will also help compare newer minimally invasive surgical therapies for BPH.^26-28^

## Conclusion

Artificial intelligence is gaining traction in urology, with the potential to improve diagnostics and patient care. The results of AI-based applications in BPH are promising but lack generalizability of results. However, in the future, we will see a shift in the clinical paradigm as AI applications will find their place in the guidelines and revolutionize the decision-making process. As AI becomes increasingly common, it is also critical to develop AI literacy among urologists.

## Figures and Tables

**Table 1.** Summary of Studies Looking at Applications of AI in Benign Prostatic Hyperplasia

**Author**	**Sample Size**	**AI Model Used**	**Objective**	**Key Findings**
Fu et al^3^	6 patients	Fuzzy systems	Initial diagnosis and evaluation of BPH and comparison with the existing methods for evaluation	This system accurately assessed even when the objective information provided by the patient was uncertain and hybrid.
Torshizi et al^15^	44 patients	Fuzzy systems	Histopathological diagnosis of BPH	The model achieved an accuracy of 90%, suggesting that decisions suggested by the fuzzy system and expert panel matched in 90% of the cases.
Khalid et al^16^	50 images, 169 regions	Computer vision-based system	Diagnose the level of severity of BPH and recommend the treatment	The model achieved an accuracy of 96.3% in correctly identifying the hyperplastic glandular areas.
Fusco et al^17^	1500 patients	Clinical data mining analysis system	To identify factors associated with clinical response to placebo or tadalafil 5 mg once daily	Highest combined sensitivity and specificity of 70% and 50%, respectively. The efficacy of tadalafil 5 mg OD in the treatment of LUTS–BPH in the majority of patients was reaffirmed.

BPH, benign prostatic hyperplasia; LUTS, lower urinary tract symptoms; OD, once daily.
